# Identification of hub subnetwork based on topological features of genes in breast cancer

**DOI:** 10.3892/ijmm.2014.2057

**Published:** 2014-12-30

**Authors:** DA-YONG ZHUANG, LI JIANG, QING-QING HE, PENG ZHOU, TAO YUE

**Affiliations:** 1 Department of Thyroid and Breast Surgery, Jinan Military General Hospital of Chinese PLA, Jinan, Shandong 250031, P.R. China; 2 Department of Prevention and Health Care, Central Hospital of Zibo City, Zibo, Shandong 255036, P.R. China

**Keywords:** hub subnetwork, gene signature, protein interaction networks, pathway, breast cancer

## Abstract

The aim of this study was to provide functional insight into the identification of hub subnetworks by aggregating the behavior of genes connected in a protein-protein interaction (PPI) network. We applied a protein network-based approach to identify subnetworks which may provide new insight into the functions of pathways involved in breast cancer rather than individual genes. Five groups of breast cancer data were downloaded and analyzed from the Gene Expression Omnibus (GEO) database of high-throughput gene expression data to identify gene signatures using the genome-wide global significance (GWGS) method. A PPI network was constructed using Cytoscape and clusters that focused on highly connected nodes were obtained using the molecular complex detection (MCODE) clustering algorithm. Pathway analysis was performed to assess the functional relevance of selected gene signatures based on the Kyoto Encyclopedia of Genes and Genomes (KEGG) database. Topological centrality was used to characterize the biological importance of gene signatures, pathways and clusters. The results revealed that, cluster1, as well as the cell cycle and oocyte meiosis pathways were significant subnetworks in the analysis of degree and other centralities, in which hub nodes mostly distributed. The most important hub nodes, with top ranked centrality, were also similar with the common genes from the above three subnetwork intersections, which was viewed as a hub subnetwork with more reproducible than individual critical genes selected without network information. This hub subnetwork attributed to the same biological process which was essential in the function of cell growth and death. This increased the accuracy of identifying gene interactions that took place within the same functional process and was potentially useful for the development of biomarkers and networks for breast cancer.

## Introduction

The occurrence of cancer generally results from the accumulation of inherited and somatic mutations in oncogenes and tumor suppressor genes. Breast cancer is characterized by a distinct metastatic pattern involving regional lymph nodes, the bone marrow, lungs and liver (1). It is thought that the incidence of breast cancer is the result of the abnormal expression of many genes (2), including cancer markers, identified after scoring the expression pattern of each gene. Although there has been extensive research on the gene markers of breast cancer, the results have not been uniform and share only a small number of genes in common (3,4). Some genes associated with breast cancer mutations are also typically not detected through the analysis of differential expression, even though they are essential in the network by interconnecting many differentially expressed genes. The importance of these genes will thus not be disclosed in the detection of individual marker genes.

Based on the shortcoming, a more effective means has adopted by combining gene expression measurements over groups of genes that fall within common pathways. This involves the identification of cancer markers by scoring known pathways and evaluating the coherency of changes in gene expression (5). However, the problem that remains is that a large number of human genes have not yet been assigned to a definitive pathway based on pathway analysis. Network-based approaches offer an effective means to at least partially solve this issue by providing potential cancer diagnostic molecular markers and connecting them.

With the development of bioinformatics analysis, network-based approaches have become more powerful and informative for the study of disease mechanisms (6). A number of researchers have suggested the detection of disease-related networks, for instance, the co-expression network (7), protein-protein interaction (PPI) network (8), protein phosphorylation networks (9) and the DNA methylation network (10). The study of these networks, particularly the study of the PPI network provides valuable information on biological systems. PPI networks are prevalent in cancer research and nonetheless studies have revealed interesting topological properties of PPI networks (11) with respect to gene essentiality. Studies (12,13) have identified subnetworks of higher accuracy as cancer markers based on the coherent expression patterns of the genes associated with a PPI network. Functional pathways or clusters may be viewed with the required subnetworks which integrate the most highly connected proteins/genes through their interactions.

In this study, we constructed a PPI network by linking causal breast cancer genes with the selected gene signatures using the genome-wide global significance (GWGS) method. Pathways and clusters were selected with enriched gene signatures using Kyoto Encyclopedia of Genes and Genomes (KEGG) pathway enrichment analysis and the molecular complex detection (MCODE) clustering algorithm, respectively. Four types of centralities of gene signatures, pathways and clusters were analyzed to obtain the hub nodes and three significant subnetworks. The hub subnetwork was formed by connecting the common hub genes with the intersection of the above three significant subnetworks. Thus, taking into consideration the genes that participated in a subnetwork whose overall activity was discriminative, this would implicate genes with a low discriminative potential (i.e., those that were not significantly differentially expressed) to increase the accuracy of identifying genetic alterations and predicting the likelihood of cancer functions with a network-based method.

## Materials and methods

### Subject samples

Microarray expression profile breast cancer biological data (E-GEOD-29431), (E-GEOD-3744) (4), (E-GEOD-42568) (14), (E-GEOD-50567) (3) and (E-GEOD-7904) were downloaded from different experimental origins using the Gene Expression Omnibus (GEO) database. A total of 281 breast cancer samples and 69 normal samples were included. Following the analysis of these data by RAM, quantiles, median polish summarization methods and unqualified chips were eliminated leaving only qualified data into the next step through quality control. The gene expression values of all data were transformed to a comparable level, a digital expression profile for further analysis.

### Detection of gene signatures

The gene signatures were screened using a novel model: genome-wide relative significance (GWRS) and GWGS with some modifications (15). The value of GWGS was applied to integrate and analyze the independent microarray studies. A gene with a large GWGS value was considered to be globally significant across multiple independent studies. GWGS was used to identify the gene signatures in breast cancer with some modifications. Briefly, gene signatures were identified by two steps: first, the GWRS of *i*-th gene in the *j*-th dataset was measured using the following formula:

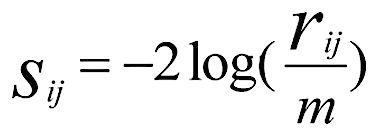


The number of datasets was denoted by *n*, the number of unique genes across *n* datasets was denoted by *m*; r_ij_ (*i* = 1-*m*, *j* = 1-*n*) was the rank number of the *i*-th gene in the *j*-th study. When a gene was mapped to multiple probe-sets, the maximum value was given to indicate the expression of the probe-set. Genefilter package (Bioconductor) was used to select genes before GWRS. The gene was removed if it was absent in one dataset. The degree of the differential expression of genes was measured by fold change. We assigned a rank number for each gene according to their differential expression.

Second, the GWGS value of the genes were measured using the following formula:

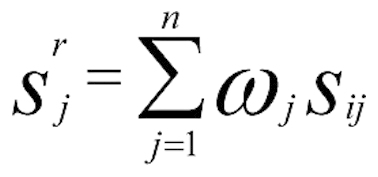
where *ω_j_* represented the relative weight of the *j*-th dataset. The value of weight can be assigned based on the data quality of the *j*-th datasets, the value of *ω_j_* can also be used to reflect the differential importance of biopsy versus cell line samples that biological scientists may wish to take into account. We assigned equal weight to each data. The P-values for all genes were recorded after being analyzed using the Linear Models for Microarray Data (Limma) 3.20.8 package, as previously described (16). The highest P-value was obtained by the maximum P-value (maxP) model which took the maximum P-value as the test statistic (17) with the intersection of the microarray datasets. The genes with |log_2_FC| >2 and P<0.01 were selected for further research.

### Construction and analysis of PPI network

The protein interaction data were selected from the Search Tool for the Retrieval of Interacting Genes/Proteins (STRING) 9.1 database and a network was constructed by linking causal disease genes with the selected gene signatures using Cytoscape 3.1.0, a free software package for visualizing, modeling and analyzing the integration of biomolecular interaction networks with high-throughput expression data and other molecular states (18).

Subsequently, we investigated the substructure of the biggest protein interaction network extracted from the above constructed network and focused on highly connected nodes known as clusters using the MCODE (19) clustering algorithm, including vertex weighting, complex prediction and optional post-processing. The core-clustering coefficient was proposed as a metric to sort the vertices in a graph with respect to their local neighborhood density. *k*-core was defined as a graph G of minimal degree *k*, where for all *v* in *G*, deg(*v*)>= *k*. At the stage of vertex weighting, all vertices based on their local network density were weighted using the highest *k*-core of the immediate neighborhood. At the stage of complex prediction, the vertex weighted graph was first taken as input and a complex with the highest weighted vertex was seeded, then recursively moved outward from the seed vertex. This included vertices in the complex whose weight is above a given threshold. The threshold is a given percentage away from the weight of the seed vertex. As a post-processing step, clusters are enhanced with additional neighborhood vertices that are members of other clusters, resulting in overlapping clusters. The software of the MCODE algorithm was obtained from http://baderlab.org/Software/MCODE. The highly interacting nodes in the clusters were identified by parameters keeping K-core = 4, node score cut-off = 0.3 and max depth up to 100.

### Centralities based analysis of complex networks

Studies have demonstrated the presence of strong correlations between the PPI network structure and the functional role of its protein/gene constituents (20,21). In order to understand the functionality of complex systems of gene signatures, we constructed the protein-protein network for gene signatures and characterized the biological importance of genes over indices of topological centrality using Cytoscape 3.1.0. We analyzed centralities related to the local (degree) scale and the global (stress centrality, betweenness centrality and closeness centrality) scale which were used to describe the importance of nodes.

Degree quantifies the local topology of each gene, by summing up the number of its adjacent genes (22). It gives a simple count of the number of interactions of a given node.

Stress centrality is considered the number of nodes in the shortest path between two other nodes; the stress is a node centrality index. Stress is calculated by measuring the number of shortest paths passing through a node. The ‘stress’ [*Cstr* (*v*)] of a node *v* is calculated as follows:

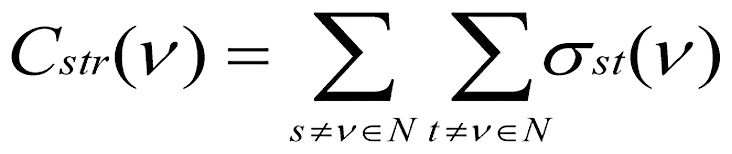
[1]

To calculate the *Cstr* (*v*) of a node *v*, all shortest paths in a graph G are calculated and then the number of shortest paths passing through *v* is counted. A ‘stressed’ node is a node traversed by a high number of shortest paths.

Betweenness centrality (23) is another topological metric in graphs for determining how the neighbors of a node are interconnected. It is considered the ratio of the node in the shortest path between two other nodes. The betweenness centrality of a node *v* is given by the expression:

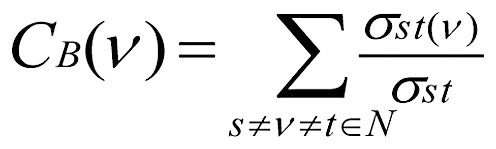
[2]

Betweenness centrality of a node scales with the number of pairs of nodes as implied by the summation indices*.* Therefore, the calculation may be rescaled by dividing the number of pairs of nodes not including *v*, so that *C_B_*(*v*) ∈ [0,1]. *σ_st_* is the total number of shortest paths from node *s* to node *t* and *σ_st_* (*v*) is the number of those paths that pass through *v* in formula 1 and 2.

Closeness centrality is a measure of the average length of the shortest paths to access all other proteins in the network (22). The larger the value, the more central is the protein. The closeness centrality, *C_c_*(*v*) was calculated for each functional category, taking into consideration all the shortest paths for each node. *C_c_*(*v*) of node n is defined as the reciprocal of the average shortest path length and is computed as follows:

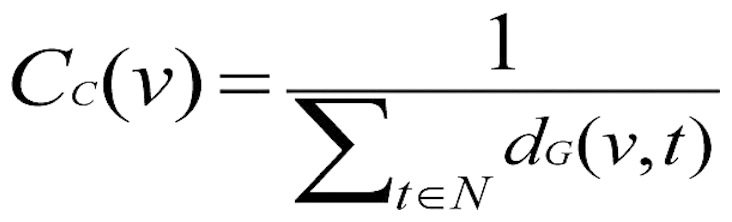
[3]where *d_G_* (*s, t*) represents the length of the shortest path between two nodes *s* and *t* in graph G, which is the sum of the weights of all edges on this shortest path. *d_G_* (*s*, s) = 0, *d_G_* (*s, t*) = *d_G_* (*t, s*) in the undirected graph.

### KEGG pathway enrichment analysis

To further investigate the signaling pathway of the selected gene signatures, we performed a pathway analysis to assess the functional relevance of selected gene signatures based on the KEGG database, a knowledge base for the systematic analysis of gene functions, linking genomic information with higher order functional information (24). It is a widely used comprehensive inference for pathway mapping of genes. The analysis of gene signatures was performed using the online tool, DAVID Bioinformatics Resources 6.7 (25). The EASE score was used to evaluate the significant categories. KEGG pathways with P-values <0.05 and 0.01 were considered to indicate statistical significance in a category.

### Statistical analysis

To compare the degree, stress centrality, betweenness centrality and closeness centrality among each cluster and each significant pathway, one-way analysis of variance (ANOVA) was employed for multiple pair-wise comparisons. A P-value was estimated for each compared pair (P<0.05, P<0.01, P<0.0001) and a P-value <0.05 was considered to indicate a statistically significant difference. Statistical analysis was performed using SPSS 17.0 software (SPSS Inc., Chicago, IL, USA)*.*

## Results

### Screening of gene signatures

**Figure 1:**
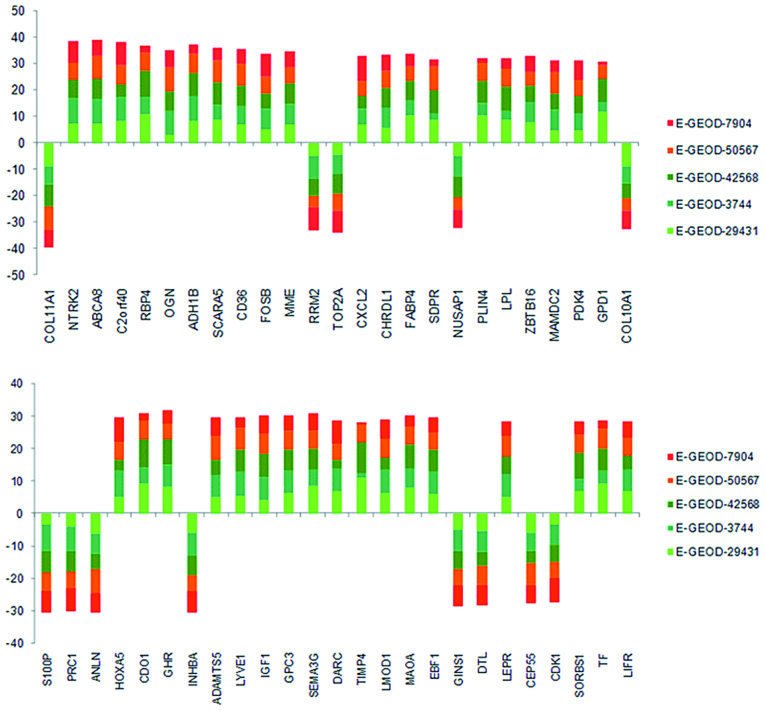
Column chart of expression changes of the top 50 ranked gene signatures in the five breast cancer data sets. Each single colored bar represents the fold change of a gene signature in a specific breast cancer data set. Bars plotted above the x-axis denote upregulation, while those plotted below the x-axis denote downregulation. The gene signatures are ordered depending on their genome-wide global significance (GWGS) values.

Five microarray datasets from different origins were integrated in the analysis to identify robust gene biomarker signatures for breast cancer using the GWGS model. A rank number for each gene according to their degree of differential expression (fold change) was obtained. A total of 20,109 genes (i.e., across all five microarray dataset intersections) were identified and the GWGS values of these genes were measured using GWGS (*S^r^_j_*) as defined above. A gene with a large *S^r^_j_* value is considered to be significant across multiple independent studies (i.e., globally significant). The log_2_FC average of common genes and highest P-values with maxP model were obtained from five datasets. The 487 genes were selected with |log_2_FC| >2 and P<0.01 as the starting point for our new proposed gene signatures, including 364 upregulated genes and 123 downregulated genes. The top 50 ranked gene signatures are listed in Table I and their degrees of differential expression are presented in Fig. 1, which were uniform for each gene among the five data sets using the GWGS method. The gene signatures corresponding to the top five GWGS value were as follows: collagen, type XI, alpha 1 (COL11A1; *S*=14.06), neurotrophic tyrosine kinase, receptor, type 2 (NTRK2; *S*=13.98), ATP-binding cassette, sub-family A (ABC1), member 8 (ABCA8; *S*=13.65), chromosome 2 open reading frame 40 (C2orf40; *S*=13.58), retinol binding protein 4 (RBP4; *S*=13.42). The 487 genes were selected for further research.

### PPI network construction and subnet analysis

**Figure 2:**
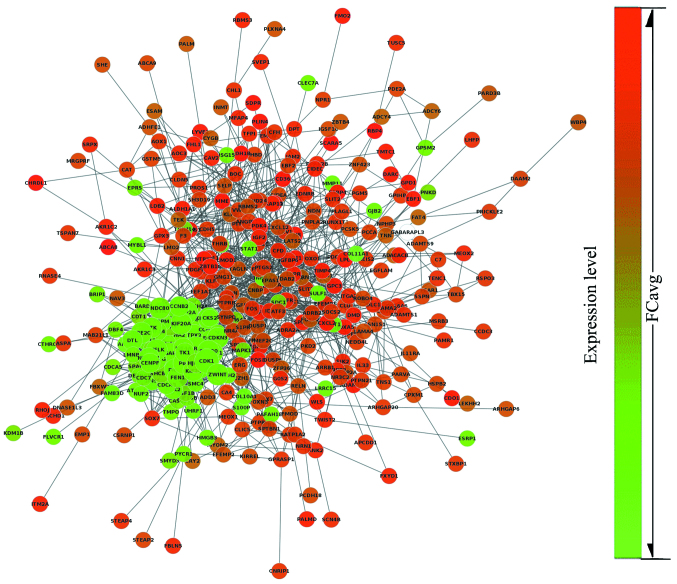
Interactome of the 366 genes showing 366 nodes and 2,760 edges in the protein-protein interaction map encompassing four clusters in breast cancer. Genes were denoted as nodes in the graph and interactions between them were presented as edges. Green color indicates downregulated genes, red color indicates upregulated genes; the node size represents the degree value.

**Figure 3:**
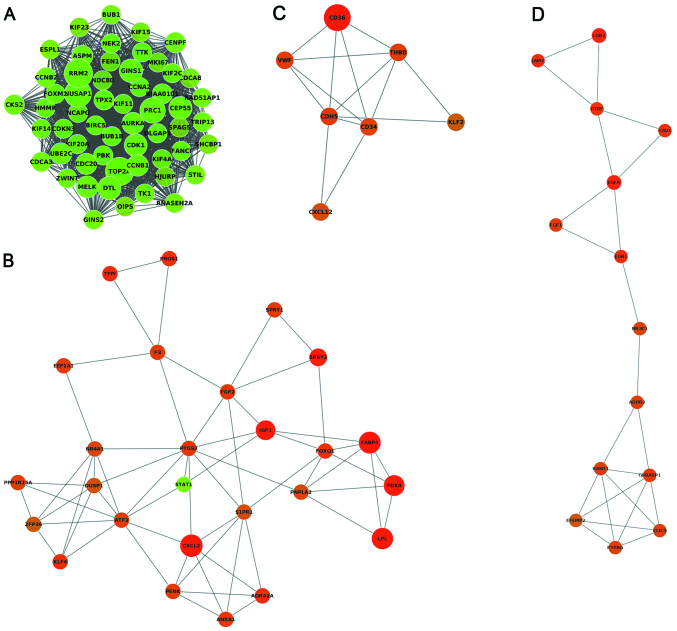
Best four interconnected clusters among the 366 genes and their interactions with neighboring genes. Green color indicates downregulated genes, while red color indicates upregulated genes; the node size represents the genome-wide global significance (GWGS) value. (A–D) cluster1, cluster2, cluster3 and cluster4, respectively.

According to the PPI dataset downloaded from STRING, the resulting breast cancer-related PPI network was composed of 442 gene signatures and 2,853 interactions. The network was binary and all interactions were unweighted and undirected. The giant component which included the majority of the entire network genes containing 366 nodes and 2,760 edges was constructed (Fig. 2) based on our analysis. The size of each node represented the degree index. The degree of its nodes indicated the number of interactions to a single node with all the other nodes. The MCODE clustering algorithm was used to identify the clusters in the PPI network. Using the MCODE plugin, the results revealed that four clusters (highly interconnected regions) (Fig. 3) in the networks were obtained with parameters set as follows: degree cut-off = 0.3, K-core = 4, max depth = 100. A cluster is a complete n-node sub-graph, which means that within a sub-graph, each pair of nodes is connected by an edge. The nodes of cluster1, cluster2, cluster3 and cluster4 were 1,437, 61, 14 and 23, respectively (Table II).

### Centralities of the networks

**Figure 4:**
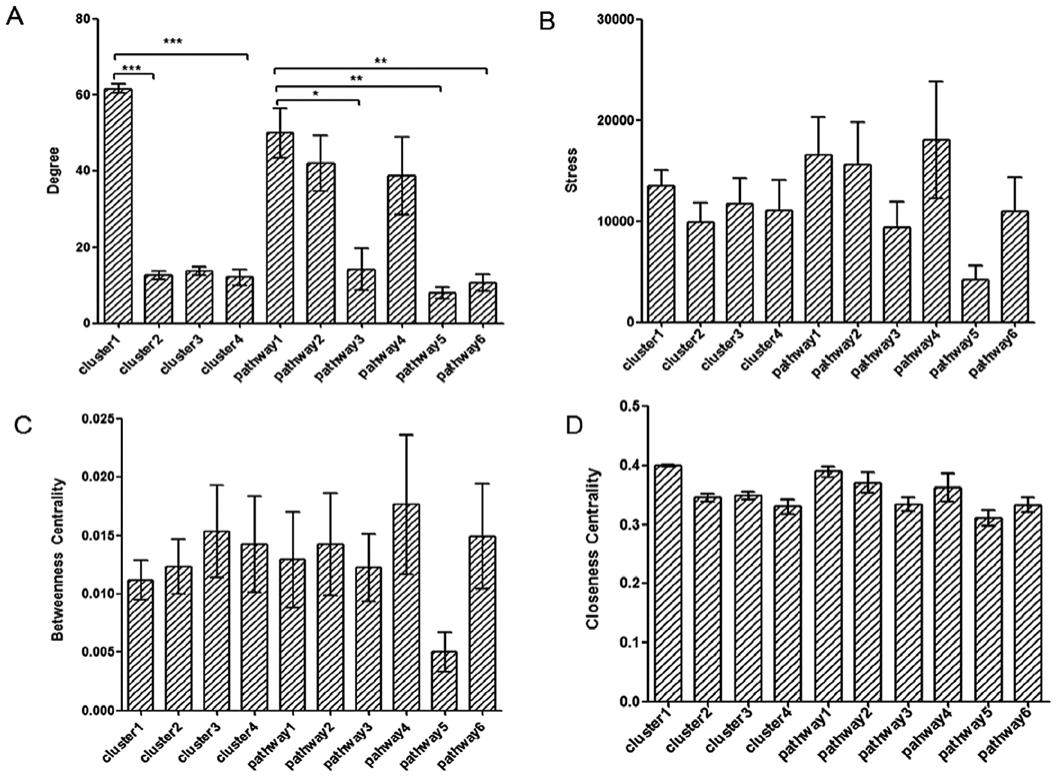
Integrated centralities based analysis of clusters and pathways. (A–D) Comparisons of degree, stress centrality, betweenness centrality and closeness centrality among the four clusters and six significant pathways, respectively. Pathway1 to pathway6: cell cycle, oocyte meiosis, ECM-receptor interaction, progesterone-mediated oocyte maturation, complement and coagulation cascades and focal adhesion, respectively. There were significant differences between cluster1 and cluster2, cluster3 of degree analysis (P<0.0001). Degree of cell cycle was significant with ECM-receptor interaction (P<0.05), complement and coagulation cascades (P<0.01), and focal adhesion (P<0.01). All four values of complement and coagulation cascades pathway were the lowest. There were no significant differences among the other groups apart from pathway5. The significant level was analyzed by one-way ANOVA. ^*^P<0.05, ^**^P<0.01 and ^***^P<0.0001.

This was used to indicate the relevance of a gene as functionally capable to hold together the communicating nodes in a biological network. The higher the value, the higher the relevance of the gene in connecting regulatory molecules. We computed four centralities for each gene in the PPI network. By assessing centrality at the local and global level of degree, stress centrality, betweenness centrality and closeness centrality, a total of 366 genes centralities were obtained and the information corresponding to the centralities of the top five ranked genes was represented, as listed in Table III. The results revealed that cyclin-dependent kinase 1 (CDK1) was the top one ranked gene; however, the results of various centralities based analyses of the same gene were not consistent. However, centralities based analysis of baculoviral inhibitor of apoptosis repeat-containing 5 (BIRC5) and epidermal growth factor receptor (EGFR) focused on ranking the top two and three. The results also revealed that the top genes as hub nodes were mostly distributed in cluster1, for instance CDK1, BIRC5, protein regulator of cytokinesis 1 (PRC1), topoisomerase II alpha (TOP2A), cyclin B1 (CCNB1), cyclin-dependent kinases regulatory subunit 2 (CKS2) and cyclin A2 (CCNA2), while EGFR was in cluster4. The overall centralities of the four subnetwork clusters were analyzed. As regards node degree distribution, cluster1 had the highest degree with 61.68 and it had significant differences with cluster2 (P<0.0001), cluster3 (P<0.05) and cluster4 (P<0.0001), as shown by one-way ANOVA. There were no significant differences between groups apart from the complement and coagulation cascades pathway in the other three global centralities based analyses (Fig. 4).

### KEGG pathway enrichment analysis

**Figure 5:**
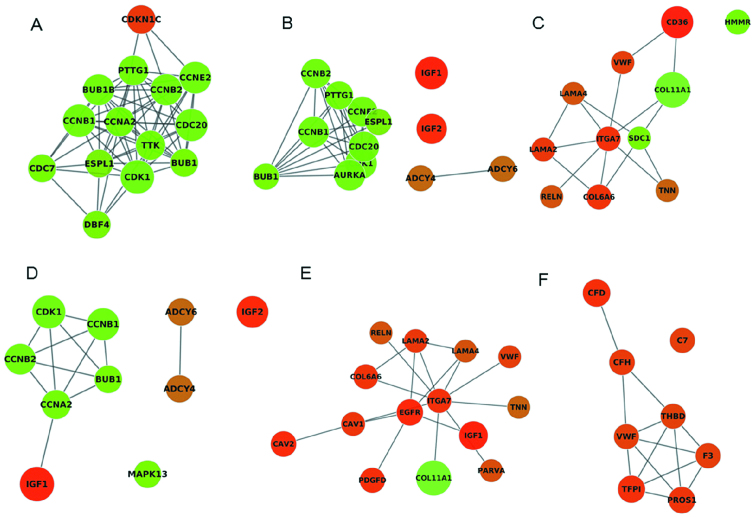
Six subnetworks constructed of significant (P<0.01) KEGG enrichment pathways of breast cancer. Nodes and links represent human genes and gene interactions, respectively. (A) Cell cycle; (B) Oocyte meiosis; (C) ECM-receptor interaction; (D) Progesterone-mediated oocyte maturation; (E) Focal adhesion; (F) Complement and coagulation cascades.

**Figure 6:**
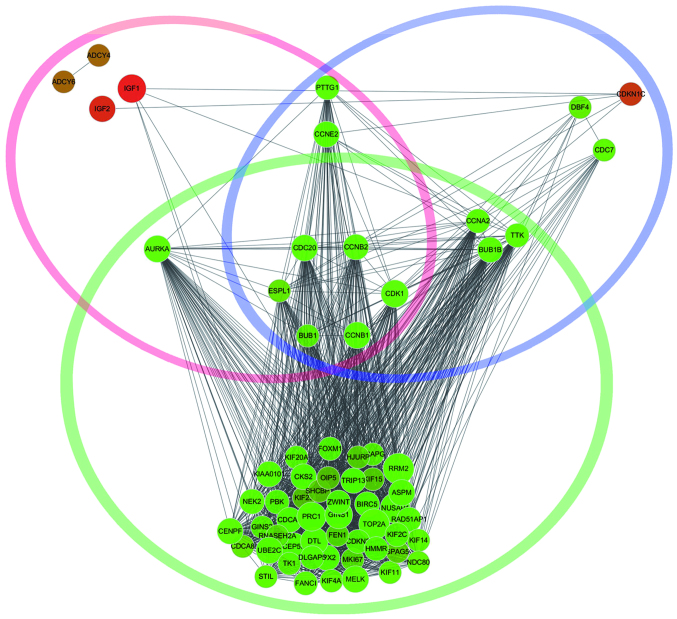
Graphical representation of the hub subnetwork composed with the significant cluster and pathways intersection. The red circle represents cell cycle, the blue circle represents oocyte meiosis, and the green circle represents cluster1. Common genes could be observed clearly between any two groups. CDK1, CCNB1, ESPL1, CCNB2, CDC20 and BUB1 as common genes among these three groups composed the hub subnetwork which was essential in the function of cell growth and death biological process.

We conducted the enrichment analysis for the 487 genes in which 25 genes were not mapped in the KEGG database. A total of 118 pathways were selected after being analyzed using the EASE method. The results revealed that 462 genes were significantly (P<0.05) enriched in 11 pathways (Table IV). The two most significant terms were cell cycle (P=1.88×10^−5^) and the oocyte meiosis (P=2.12×10^−5^) pathway which were related to cell growth and death, which included 15 and 14 genes, respectively. In addition, six pathways with a significance level of P<0.01 were established as subnetworks, which were the cell cycle, oocyte meiosis, ECM-receptor interaction, progesterone-mediated oocyte maturation, complement and coagulation cascades and focal adhesion (Fig. 5). The centralities of these pathways were analyzed (Fig. 4) by aggregating the centralities of all genes enriched in one pathway (or a functional subnetwork); the degree of cell cycle containing 14 genes was found to be significant with ECM-receptor interaction (P<0.05), complement and coagulation cascades (P<0.01) and focal adhesion (P<0.01). Although there was no significance in the comparison between stress centrality, betweenness centrality and closeness centrality of these six pathways, it was easy to observe that the values of these centralities of the cell cycle were higher, which may be viewed as a putative marker for participating in breast cancer with functional insight. Besides, we found that CDK1, CCNB1, extra spindle pole bodies homolog 1 (*S. cerevisiae*) (ESPL1), CCNB2, CDC20 and BUB1 were commom genes in significant cluster1, cell cycle and oocyte meiosis (Fig. 6). The cell cycle and oocyte meiosis pathway, with common function, were contacted effectively by cluster1 using the PPI network and were presented visually in the form of a diagram.

## Discussion

In this study, we aimed to identify a hub subnetwork with functional insight associated with cell growth and death using a protein-network-based approach. A total of 487 gene signatures were selected using the GWGS method from five sets of breast cancer data and the changes in gene expression were measured clearly both with fold change criterion and GWGS values. With 422 gene signatures mapped from the STRING database, a giant component PPI network was constructed with 366 nodes. After applying the MCODE clustering algorithm and KEGG pathway enrichment analysis, four clusters with highly connected nodes and six significant (P<0.01) pathways were obtained, respectively. The degrees and three types of centralities related to the global scale were analyzed for all the detected genes and the significant complex (i.e., four clusters and six pathways). The top five ranked genes as hub nodes and one cluster (cluster1), two pathways (cell cycle and oocyte meiosis) as significant groups with high degrees and centralities were identified. We found that almost all hub nodes existed in significant cluster1 which connected the cell cycle and oocyte meiosis pathways effectively. It was found that CDK1, CCNB1, ESPL1, CCNB2, CDC20 and BUB1, some of the top ranked genes, composed a small sized hub subnetwork attributing to the biological processes of cell growth and death.

The capabilities of bioinformatics tools for the detection of differential gene expression, network analysis, gene ontology and gene-disease relationships (26,27) together with all available data on protein/gene expression during breast cancer provide an interesting and valuable opportunity for the study of diseases. At present, many gene signatures have been identified based on fold change criterion to assess differential expression (28). In our study, the degree of change in gene expression was also clearly shown using the GWGS method. However, although there have been numerous studies on gene signatures of breast cancer, the results have not been uniform (3,4). For example, Berlingieri *et al* (29) found that UbcH10 was overexpressed in a variety of tumor tissues in breast cancer, lung cancer and colon cancer, and that its high expression was closely related to tumor occurrence, development metastasis and the degree of malignancy. Rutnam *et al* (30) demonstrated that FN1 or cell adhesion changes was a key step in malignant transformation, and that it may prevent malignant or confine cancerous lesions to the epithelium by regulating FN1. Thus, it is still prudent further detect essential genes after identifying gene signatures. Besides, it may not work effectively in different datasets even though the gene signatures were the same in some studies (31). However, the results of fold change were uniform (i.e., either all were upregulated, or downregulated) from the five breast cancer data of the identified gene signatures by combining the GWGS and maxP methods in our research.

Networks as a powerful tool have attracted a great deal of attention in the analysis of many biological and communication systems. Protein interaction network analysis provides an effective method for estimating and understanding the likelihood of the existing yet unknown connections between proteins/genes (32). It can provide significant instructions for mining unknown connections in incomplete networks. However, in PPI networks, although the data of large-scale protein interaction are accumulated with the development of high throughput testing technology, a certain number of interactions are not tested, which may be very important. This issue has been resolved to some extent using clustering methods which have previously been shown to be useful in identifying protein/gene interactions that take place within the same cellular process (33). In this study, we applied the MCODE clustering algorithm to explore gene-gene connectivity in a more informative manner and obtained four clusters with highly connected nodes.

In many PPI networks, essentiality is correlated with the topological placement of the proteins/genes in the network, and while connectivity provides an indication of the importance of a gene, it is possible to further classify the topological role of highly connected genes based on their locality. That is, hubs that are highly connected in a PPI network tend to correspond to essential genes (34). In this study, topological analysis of all detected genes and the significant clusters and pathways was carried out through stress centrality, closeness centrality, betweenness centrality and node degree distribution. The top five ranked genes were identified. Moreover, we identified the topologically related pathways and processes. These pathways were unlikely to be compared using traditional term-based analysis. In our results, cluster1, the cell cycle and oocyte meiosis pathways with high centralities were considered significant compared with the other groups. The hub subnetwork composed of these three significant groups and intersecting genes was presented visually and was shown to participate in cell growth and death processes. Our data provide functional insight into the identification of hub subnetworks which may play a vital role in the progression of breast cancer.

## Figures and Tables

**Table I:** The 487 gene signatures identified using the genome-wide global significance (GWRS) method and the values of the top 50 genes.

Gene	GWGS	Gene	GWGS	Gene	GWGS	Gene	GWGS
COL11A1	14.06	CXCL2	11.71	PRC1	10.74	TIMP4	10.14
NTRK2	13.99	CHRDL1	11.55	ANLN	10.73	LMOD1	10.14
ABCA8	13.65	FABP4	11.45	HOXA5	10.64	MAOA	10.10
C2orf40	13.58	SDPR	11.37	CDO1	10.60	EBF1	10.08
RBP4	13.42	NUSAP1	11.32	GHR	10.56	GINS1	9.96
OGN	13.13	PLIN4	11.22	INHBA	10.52	DTL	9.91
ADH1B	13.11	LPL	11.22	ADAMTS5	10.50	LEPR	9.90
SCARA5	12.45	ZBTB16	11.13	LYVE1	10.46	CEP55	9.85
CD36	12.19	MAMDC2	11.03	IGF1	10.43	CDK1	9.74
FOSB	11.99	PDK4	10.97	GPC3	10.33	SORBS1	9.74
MME	11.91	GPD1	10.93	SEMA3G	10.33	TF	9.70
RRM2	11.87	COL10A1	10.93	DARC	10.17	LIFR	9.69
TOP2A	11.79	S100P	10.80				

**Table II:** The clusters generated by the molecular complex detection (MCODE) clustering algorithm at K-core = 4, node score cutoff = 0.3 and max depth up to 100 along with interacting gene partners.

Cluster name	Score	Nodes	Edges
1	52.255	56	1,437
2	4.88	26	61
3	4.667	7	14
4	3.538	14	23

**Table III:** Centralities based analysis and the values of the top five ranked genes.

No.	Terms	Value	Terms	Value	Terms	Value	Terms	Value
	Degree		Stress		Betweennes centrality		Closeness centrality	
1	CDK1	81	CDK1	55156	CDK1	0.0559	CDK1	0.4416
2	BIRC5	80	BIRC5	42696	EGFR	0.0529	BIRC5	0.4333
3	CCNA2	79	EGFR	41584	BIRC5	0.0440	CCNA2	0.4248
4	TOP2A	74	FOS	40114	FOXO1	0.0424	CCNB1	0.4218
5	PRC1	73	CKS2	38250	FOS	0.0406	KIAA0101	0.4218

**Table IV:** Eleven significant (P<0.05) KEGG pathways.

Term	Count	P-value	Genes
Cell cycle	15	1.88E-05	CDC7, CDK1, DBF4, TTK, CDC20, ESPL1, PTTG1, CCNB1, CDKN1C, CCNE2, CCNB2, MAD2L1, BUB1, BUB1B, CCNA2
Oocyte meiosis	14	2.12E-05	ADCY4, CDK1, ADCY6, IGF1, AURKA, CDC20, ESPL1, IGF2, PTTG1, CCNB1, CCNE2, CCNB2, MAD2L1, BUB1
ECM-receptor interaction	11	1.85E-04	LAMA2, VWF, LAMA4, SDC1, CD36, COL6A6, ITGA7, TNN, RELN, COL11A1, HMMR
Progesterone-mediated oocyte maturation	11	2.25E-04	CCNB1, CDK1, ADCY4, MAD2L1, CCNB2, MAPK13, ADCY6, BUB1, IGF1, IGF2, CCNA2
Complement and coagulation cascades	8	0.004395	VWF, C7, THBD, F3, CFH, TFPI, CFD, PROS1
Focal adhesion	14	0.007100	EGFR, CAV2, CAV1, IGF1, LAMA2, VWF, LAMA4, COL6A6, ITGA7, RELN, TNN, PDGFD, COL11A1, PARVA
Aldosterone-regulated sodium reabsorption	5	0.033673	NR3C2, IGF1, IGF2, NEDD4L, ATP1A2
Pathways in cancer	17	0.036557	EGFR, PTGS2, EPAS1, TGFBR2, RUNX1T1, FOXO1, IGF1, BIRC5, ZBTB16, MECOM, STAT1, CCNE2, LAMA2, FOS, LAMA4, FGF1, FGF2
Prostate cancer	7	0.050592	EGFR, CCNE2, IGF1, FOXO1, CREB5, IGF2, PDGFD
p53 signaling pathway	6	0.052858	CCNE2, CCNB1, CDK1, CCNB2, RRM2, IGF1
Ether lipid metabolism	4	0.086840	ENPP2, PAFAH1B3, PPAP2A, PPAP2B
